# Retraction: Glioma-Derived ADAM10 Induces Regulatory B Cells to Suppress CD8^+^ T Cells

**DOI:** 10.1371/journal.pone.0249104

**Published:** 2021-03-19

**Authors:** Zhuo-peng Ye, Hai-yong He, Hui Wang, Wen-sheng Li, Lun Luo, Zhen-chao Huang, Ying Guo, the PLOS ONE Editors

After this article [1] was published, concerns were raised about results reported in Fig 2.

Specifically:

- Similarities were noted between the following data shown in Fig 2B. These include image similarities in the background areas within the indicated lanes.
  ○ In the TGF-beta blot, lanes 1 and 2 appear similar to lanes 7 and 8, respectively.
  ○ In the β-actin blot, the background speck patterns in lanes 5 and 6 appear similar to those in lanes 8 and 7, respectively, when flipped horizontally.
- In the blot images shown in Figs 2A, 2C, and 2D, similarities were noted between areas of the background above and below the bands.

In response to the above concerns, the authors noted that the wrong files were used in compiling the figure and that the original data supporting Fig 2 are no longer available. They offered replacement data from replication experiments, but the data provided did not resolve the concerns about the published figure. The corresponding author requested retraction.

In light of the above concerns, the authors and *PLOS ONE* Editors retract this article.

YG relayed that all authors agree with retraction and apologize for the issues with the published article.
